# A Pilot Comparative Study of 26 Biochemical Markers in Seminal Plasma and Serum in Infertile Men

**DOI:** 10.1155/2015/805328

**Published:** 2015-10-11

**Authors:** Rui-Xiang Feng, Jin-Chun Lu, Hong-Ye Zhang, Nian-Qing Lü

**Affiliations:** ^1^Department of Laboratory Science, Nanjing Hospital, Jiangsu Corps, The Armed Police Force, PLA, Nanjing 210028, China; ^2^Department of Laboratory Science, Nanjing Hospital Affiliated to Nanjing Medical University, Nanjing 210006, China; ^3^Jiangsu Family Planning Research Institute, Nanjing 210036, China

## Abstract

*Introduction*. The relationships of the biochemical components in seminal plasma and serum, and their origins and physiological effects in male reproductive system have been poorly understood. *Methods*. Based on the calibration and quality control measures, 26 biochemical markers, in seminal plasma and serum samples from 36 male infertility patients with nonazoospermia were detected and compared. *Results*. Only PA was undetectable in all seminal plasma samples. There were significant differences of all other 24 biochemical markers in seminal plasma and serum (*P* < 0.05) except for UA (*P* = 0.214). There were rich proteins in seminal plasma, and globulin accounted for about 90%. There were also abundant enzymes in seminal plasma, and the activities of ALT, AST, AKP, GGT, LDH, CK, and *α*HBDH in seminal plasma were significantly higher than those in serum while ADA was inversely lower. There were relatively low levels of Glu, TG, TC, and hsCRP in seminal plasma, but Glu was undetectable in 8 of 36 cases. *Conclusions*. The differences of the levels of biochemical markers in seminal plasma and serum might be associated with the selective secretion of testis, epididymis and male accessory glands, and the specific environment needed for sperm metabolism and function maintenance.

## 1. Introduction

Currently, alpha-glucosidase, acid phosphatase, zinc, and fructose levels in seminal plasma have been determined in clinical andrology laboratories to evaluate the secretion function of male accessory glands [1–3]. Like serum, seminal plasma consists of rich biochemical components. It has been shown recently that seminal plasma proteins could serve as important biomarkers for male infertility [4]. In addition, functional proteomic analysis revealed that proteins are over- or underexpressed in the seminal plasma of men with poor semen quality [5]. However, the origins of these components and their correlations with those in serum are unclear. It is also unknown whether these components could be used to evaluate male fertility. Several studies have been done to compare the levels of biochemical markers in seminal plasma and serum, most of these studies focused on animal reproduction, and investigations on biochemical markers were limited. Moreover, the origins and potential physiological effects of biochemical components in seminal plasma have been still poorly understood; thus, we designed this study to detect the levels of 26 kinds of biochemical markers in seminal plasma and serum on the basis of quality control for each marker, and all the data were compared and analyzed.

## 2. Materials and Methods

### 2.1. Reagents

Kits for the determinations of total protein (TP), albumin (Alb), alanine aminotransferase (ALT), aspartate aminotransferase (AST), alkaline phosphatase (AKP), and calcium (Ca) (Biosino Bio-Technology and Science Inc., Beijing, China), prealbumin (PA), high-sensitive C-reactive protein (hsCRP), gamma-glutamyl transpeptidase (GGT), lactate dehydrogenase (LDH), urea (Ur), creatinine (Cr), uric acid (UA), glucose (Glu), creatine kinase (CK), and adenosine deaminase (ADA) (Ningbo Medical System Biotechnology Co., Ltd., Ningbo, China), triglyceride (TG) and total cholesterol (TC) (Shanghai Zhicheng Biotechnology Co., Ltd., Shanghai, China), alpha-hydroxybutyrate dehydrogenase (*α*HBDH) (Zhejiang Dongou Diagnostics Co., Ltd., Wenzhou, China), magnesium (Mg) and phosphorus (P) (Shanghai Fosun Long March Medical Science Co., Ltd., Shanghai, China), and potassium (K^+^), sodium (Na^+^), and chlorine (Cl^−^) (Nanjing Panstar Electronics Instruments Co., Ltd., Nanjing, China) are commercially available from the abovementioned suppliers. Calibration and quality control products were provided by Randox Laboratories Ltd., Northern Ireland, United Kingdom.

### 2.2. Instruments

Olympus AU400 Automatic Biochemical Analyzer (Olympus Optical Co., Ltd., Tokyo, Japan), PSD-16a Electrolyte Analyzer (Nanjing Panstar Electronics Instruments Co., Ltd., Nanjing, China), and TGL-16B High Speed Centrifuge (Shanghai Anting Scientific Instrument Factory, Shanghai, China) were used to determine biochemical markers in this study.

### 2.3. Samples

All seminal plasma and serum samples were collected in the clinics of the Department of Reproduction and Genetics, Jinling Hospital, School of Medicine, Nanjing University, Nanjing, China. Thirty six (36) infertility patients with nonazoospermia for over 1 year, aged from 26 to 33 years, who self-reported no other disorders, were included in this study. After abstinence for 2 to 7 days, semen samples were collected by masturbation, and for serum samples collection, fasting venous blood was drawn. Semen samples were placed at 37°C for liquefaction, followed by routine semen analysis, and the remaining semen samples were centrifuged at 12 000 ×g for 5 minutes. The upper layer seminal plasma was collected for the determinations of biochemical markers. Blood samples were centrifuged at 3 000 ×g for 5 minutes to isolate serum for the same analyses as for seminal plasma. All semen samples of nonliquefaction and volume less than 1.5 mL were excluded to avoid potential influence on the accuracy of sampling [6] and ensure all biochemical markers to be detected.

### 2.4. Biochemical Analysis

First, the calibration and the determination of quality control products for all biochemical markers were performed. After all results of quality control products were within the permitted ranges, the concentrations of K^+^, Na^+^, and Cl^−^ in seminal plasma and serum specimens were detected with a PSD-16a Electrolyte Analyzer and all other biochemical markers determined with Olympus AU400 Automatic Biochemical Analyzer. The samples outside of the linearity of the method and instrumentation were diluted to obtain effective results. All serum samples were directly determined without any dilution, while the measurements of LDH, CK, *α*HBDH, K^+^, Ca, and Mg in seminal plasma were conducted after 1 : 5 dilution, and 1 : 25 dilution for GGT and P. The seminal plasma samples for the measurements of LDH, CK, *α*HBDH, Ca, Mg, GGT, and P were diluted with normal saline, and that for K^+^ was diluted with deionized water.

### 2.5. Statistical Analysis

All data were saved in the Excel tables and analyzed with the SPSS 11.0 software (SPSS Inc., Chicago, USA). The values of biochemical markers in seminal plasma and serum were presented as mean ± standard deviation and compared by paired *t*-test, and *P* < 0.05 was considered as significant difference. The correlation of biochemical markers in seminal plasma and serum was analyzed with Pearson analysis, and *P* < 0.05 was considered as significant correlation.

## 3. Results

**Figure 1:**
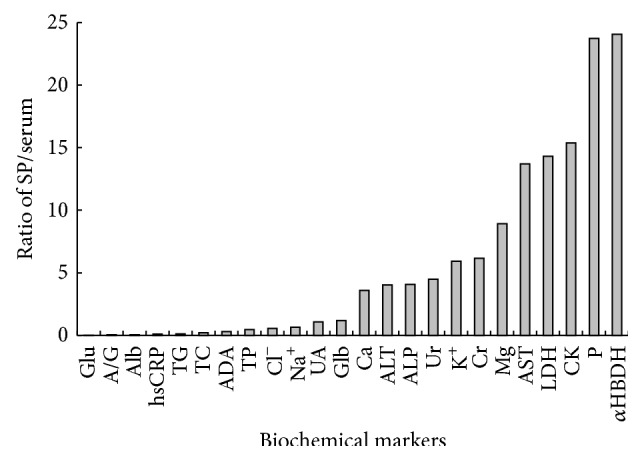
Ratios of 24 seminal plasma (SP) and serum biochemical markers except PA (undetectable) and GGT (the ratio of SP/serum up to 543.54).

The comparison of the results of biochemical markers in seminal plasma and serum was shown in Table 1. Among all 26 kinds of biochemical markers, PA were undetectable, and there was no significant difference of UA in seminal plasma and serum (*P* = 0.214), but all the results of other 24 markers in seminal plasma and serum had significant difference (*P* < 0.05). The levels of Glb, ALT, AST, ALP, GGT, LDH, Ur, Cr, CK, *α*HBDH, K^+^, Ca, Mg, and P in seminal plasma were significantly higher than those in serum, while the levels of TP, Alb, A/G, ADA, Glu, TG, TC, Na^+^, Cl^−^, and hsCRP in seminal plasma were significantly lower than those in serum (Figure 1). The level of seminal plasma Glu in 8 out of 36 cases was undetectable. The correlation analysis for 24 kinds of biochemical markers except PA (undetectable in seminal plasma) and A/G (with the same value) showed no obvious correlation of biochemical markers except significant positive correlations of UA (*r* = 0.513, *P* < 0.01) and hsCRP (*r* = 0.861, *P* < 0.01), significant negative correlation of Glu (*r* = −0.356, *P* < 0.05), and some negative correlation of TC (*r* = −0.328, *P* = 0.051) in seminal plasma and serum.

## 4. Discussion

Over the past seven decades, seminal plasma have been extensively studied. However, the comparison of biochemical markers in seminal plasma and serum has been poorly documented. So far, up to 19 biochemical markers have been reported, and the seminal plasma samples were obtained from 6 patients with spinal cord injury and 6 volunteers [7]. Rosecrans et al. [8] reported 16 biochemical markers, including Ca, Mg, K^+^, Na^+^, Cl^−^, Zn, P, glycerylphosphorylcholine (GPC), carnitine, fructose, UA, acid phosphatase (ACP), AKP, AST, LDH, and ALT in seminal plasma and serum from 24 volunteers, and their results showed that the levels of all biochemical markers except UA between seminal plasma and serum were significantly different (*P* < 0.05). Our results presented in this study were similar to them. Rosecrans et al. [8] also reported that there were significant correlations of K^+^ (*r* = 0.51), carnitine (*r* = 0.54), and AST (*r* = 0.70) in seminal plasma and serum, but we have not observed such phenomena in present study. Other comparative studies on biochemical markers in seminal plasma and serum were focused mostly on a single marker, such as the level of Mg in seminal plasma and serum samples obtained from normal fertile men and patients with premature ejaculation [9], and the levels of Ca and Mg in seminal plasma and serum from 113 men [10]. Moreover, the levels of protein, electrolytes, enzymes, and other components in seminal plasma samples in animals such as brown bears [11], rabbits [12], stallion [13], and bactrian camels [14] have been investigated, but the useful information was very limited in these studies.

In present study, we investigated the levels of the other 26 biochemical markers in seminal plasma except alpha-glucosidase, acid phosphatase, fructose, and zinc, which had been extensively evaluated for their standardized operation and quality control [1–3]. In order to avoid potential influence of particles in semen, such as sperm, lecithin body on the level, and the determination method of biochemical components in seminal plasma, seminal plasma was isolated from semen samples by centrifugation at 12 000 ×g for 5 minutes. The results of preliminary experiments for such seminal plasma samples showed good repeatability (CV < 5%). In previous studies [1–3], we have shown that there were still many spermatozoa in some seminal plasma samples obtained at 3 000 ×g centrifugation for 15 minutes. So, the inconsistency for some previously reported results may be due to the sperm residue in seminal plasma. Second, we obtained the results of all 26 markers from samples with the volume above 1.5 mL and ensured the accuracy of all results for the calibration and quality control measures made. All these efforts provided a guarantee for drawing meaningful conclusions.

Similar to serum, seminal plasma is composed of various components, and each of them has physiological significance. von Wolff et al. [15] reported that the injection of cryopreserved seminal plasma into the cervix and the posterior fornix of the vagina just after follicle aspiration in IVF or intracytoplasmic sperm injection (ICSI) treatment cycles has the potential to improve pregnancy rate. Moreover, seminal plasma was important for sperm metabolism, the maintenance of sperm function, and the survival and transport of sperm in the female reproductive tract. Our results showed that despite the fact that the UA levels in seminal plasma and serum were comparable, the other markers were significantly different in seminal plasma and serum, suggesting that the origins of the biochemical components in seminal plasma may be the secretion of epithelial cells in the reproductive tract, Sertoli cells, and spermatogenic cells in the male reproductive system for the existence of blood-testis, blood-epididymis barrier and other barriers.

Over 2 545 unique proteins in seminal plasma have been identified [16]. In our studies, TP level in seminal plasma was 33.91 ± 6.99 g/L, close to the level of 37.9 ± 5.606 g/L reported by Verdejo et al. [17]. Unlike serum, the dominant proteins in seminal plasma were globulins, which accounted for 90% of TP, while albumin accounted for about 10%. Previous studies [7, 18–21] described the possible role of albumin in seminal plasma. However, there were abundant globulins in seminal plasma, and their roles in male reproduction have been poorly understood. Moreover, in contrast to the previously reported results [22], our findings showed that PA was undetectable in seminal plasma.

A great number of enzymes in seminal plasma have been investigated. Guerin et al. [23] reported that the existence mode of enzymes in seminal plasma was similar to that in sperm, indicating that the enzymes in seminal plasma might play a role in ensuring the normal metabolism of sperm. Our results showed that the activity of ALT and AST was 76.75 ± 24.29 U/L and 439.31 ± 110.57 U/L, respectively; both of them were significantly higher than those in serum; in particular AST activity in seminal plasma was 13.71 times higher than that in serum, similar to the result reported by Hirsch et al. [7], that is, ALT: 62.3 ± 25.7 IU/L and GOT: 412 ± 191.1 IU/L. Mortimer and Bramley [24] also reported that the level of AST in seminal plasma was about 15 times higher than that in serum. Moreover, as reported by Dhami and Kodagali [25], the activity of ALT was positively correlated with that of AST (*r* = 0.514, *P* = 0.001). Whether ALT and AST in seminal plasma were derived from testis, epididymis, prostate, or seminal vesicle [7, 13, 26] was uncertain. In general, high activity of ALT and AST in seminal plasma might have some adverse effects on sperm function [25, 27–30], which may be used as a marker to evaluate sperm quality. However, further studies are needed to analyse the origins of the high levels of ALT and AST in seminal plasma, and we might speculate that some of damaged sperm or epithelial cells in the reproductive tract contribute to such elevated ALT and AST levels in seminal plasma.

GGT and AKP are two important enzymes that reflect hepatobiliary function. Our results showed that the levels of AKP and GGT in seminal plasma were 311.33 ± 337.10 U/L and 12133.33 ± 4278.56 U/L, respectively, and 4.07 and 543.54 times higher than that in serum. Chen et al. [31] reported that GGT in seminal plasma was mainly from prostate, which could be used to evaluate the secretion function of prostate instead of ACP, and that the accuracy of seminal plasma GGT detection was superior to that of ACP. However, studies on the detection of AKP in human seminal plasma were limited [7]. In boar [32] and stallion [13], the level of AKP in seminal plasma was significantly correlated with semen volume and sperm concentration. In bull [33], after semen was frozen and thawed, the level of AKP in seminal plasma increased significantly with sperm motility and fertilization rate decreasing, indicating that AKP in seminal plasma might come from the secretion of reproductive tract epithelial cells and the release of damaged sperm. However, the source of AKP in seminal plasma and its role in male reproduction need to be further investigated.

Our results also showed that LDH, CK, and *α*HBDH were rich in seminal plasma, up to 14.32, 15.37, and 24.06 times higher than those in serum, respectively. There were also some ADA in seminal plasma. The level of LDH in seminal plasma was similar to those in other published results [7, 17, 34]. It was well known that lactic acid could be converted to pyruvic acid catalyzed by LDH, which played a role in the production of ATP in sperm mitochondria [35]. Similarly, there was CK in sperm [36, 37], which was very important for sperm energy metabolism. Such high LDH and CK concentrations in seminal plasma might suggest that LDH and CK could be used as the marker to predict whether sperm energy metabolism is normal. Therefore, it is necessary to further analyse their origins and potential physiological functions. Recently, a PubMed search showed that the detection of *α*HBDH and ADA in seminal plasma has not been found in MEDLINE database, and their possible physiological roles in male reproduction remain unclear.

The results in the determination of TG, TC, Glu, UA, hsCRP, Ur, and Cr in seminal plasma showed that the level of UA was similar to that in serum, while the levels of Ur and Cr were significantly (4.49 and 6.15 times) higher than those in serum, respectively. In contrast, the levels of TG and TC in seminal plasma were only about 1/5 of those in serum, and hsCRP was only about 1/10 of that in serum, and the level of Glu in seminal plasma was extremely low, even undetectable in 8 out of 36 cases, which may be due to the fact that fructose is the main energy source for sperm capitation.

It was shown that the level of UA in seminal plasma from normal fertile men was significantly higher than that from patients with azoospermia, infertility, or the vasectomized [38]. Moreover, the level of UA in seminal plasma was significantly correlated with sperm count [38] and the percentage of sperm with normal morphology [39]. All these reported findings indicated that the UA in seminal plasma might come from testis and/or epididymis and play an important role in protecting sperm from the damage of free radicals [40]; thus, UA could be used as an antioxidation marker in clinical diagnosis. In addition, investigations on Ur, Cr [7, 38], and hsCRP [41] in seminal plasma were very limited; thus meaningful information was not available. At least three questions remain unclear: (1) whether the hsCRP in seminal plasma was a kind of stress protein in male reproductive system, (2) whether the Ur and Cr in seminal plasma were the products of sperm metabolism, and (3) what their possible physiological function and clinical significance were.

There was rich cholesterol in human sperm cell membrane, which played an important role in maintaining the normal function of sperm. It was shown that there were exchanges of cholesterol and phospholipids between sperm and seminal plasma [42]. Therefore, it was possible that the level of cholesterol in seminal plasma would lead to the disorder of the cholesterol in sperm cell membrane. Although Meseguer et al. [43] reported no significant correlation of the concentration of cholesterol in seminal plasma with the level in sperm cell membrane, Cross [44] revealed that the medium with rich cholesterol could inhibit the acrosome reaction induced by progesterone. All these observations suggested that the extremely high or low concentration of cholesterol in human seminal plasma could affect the exchange of cholesterol between sperm and seminal plasma, and the detection of cholesterol in sperm cell membrane may be more meaningful than that in seminal plasma.

There were many reports about the detection of electrolytes in seminal plasma, but the results were not entirely consistent, which might be related to the different detection methods. Our results showed that the levels of Na^+^ and Cl^−^ in seminal plasma were about 1/2 to 2/3 of them in serum, while the levels of K^+^, Ca, Mg, and P in seminal plasma were significantly higher than them in serum, especially for P up to 23.73 times. Moreover, there were significantly positive correlations between the levels of Na^+^ and Cl^−^ (*r* = 0.654, *P* = 0.000) and between Ca and Mg (*r* = 0.930, *P* = 0.000). In general, there were parallel concentrations of Na^+^ and Cl^−^ in seminal plasma, and both were significantly higher than those of K^+^. In contrast, the concentration of K^+^ in sperm was significantly higher than that of Na^+^ [45].

It was shown that the levels of Na^+^, Ca, and Mg in seminal plasma were positively correlated with the percentage of motile sperm [46, 47], while that of K^+^ was inversely correlated [47], indicating that K^+^ in seminal plasma might inhibit sperm motility, but Na^+^ promotes it. There were specific ion channels in sperm cell membrane for transferring K^+^, Na^+^, and Ca^2+^ into sperm or seminal plasma; accordingly sperm membrane potential changed and sperm motility promoted [48]. Therefore, the levels of electrolyte ions in seminal plasma should have an optimal range, and the increase and decrease of these ions will lead to the disorders of sperm motility [49]. However, whether these ions can be used to evaluate sperm motility needs to be confirmed.

There were very few reports about the detection of P in seminal plasma. Adamopoulos and Deliyiannis [50] found that there was higher level of P in seminal plasma in the patients with asthenospermia and lower level in patients with azoospermia compared with the males with normal sperm and that the level of P was positively correlated with that of fructose in patients with asthenospermia, oligospermia, or azoospermia, indicating that the origin of P in seminal plasma might be the secretion of seminal vesicle, and the detection of P in seminal plasma might reflect the secretion function of seminal vesicle.

In summary, among all the 26 biochemical markers we studied, only the level of UA was no significant difference in seminal plasma and serum. There were rich proteins in seminal plasma, but unlike serum, globulin was the dominant protein. There were also rich enzymes in seminal plasma, and the activities of ALT, AST, AKP, GGT, LDH, CK, and *α*HBDH except ADA in seminal plasma were significantly higher than those in serum. There were low levels of Glu, TG, TC, and hsCRP in seminal plasma, Glu was undetectable in 8 out of 36 cases, and PA was undetectable in all subjects. At present, the causes leading to the difference of these markers in seminal plasma and serum were unclear, due to the selective secretion of testis, epididymis and male accessory glands, and the specific environment required for sperm metabolism and function maintenance. The limitation of this pilot comparative study was relatively small sample size. Thus, further investigations should be focused on the identification of the origins and potential roles of these biochemical components in male reproduction, and comparison of the difference between fertile and infertile men based on bigger sample size.

## Figures and Tables

**Table 1:** Comparison of biochemical parameters in seminal plasma and serum (*n* = 36).

Parameters	Seminal plasma (SP)	Serum	Ratio (SP/serum)	Correlation (*r*)
Total protein (TP) (g/L)	33.91 ± 6.99	74.67 ± 3.83^*∗*^	0.46 ± 0.09	0.063
Albumin (Alb) (g/L)	2.87 ± 0.51	48.30 ± 1.93^*∗*^	0.060 ± 0.011	0.112
Globulin (Glb) (g/L)	31.05 ± 6.86	26.37 ± 3.09^*∗*^	1.19 ± 0.27	0.166
Albumin/globulin (A/G)	0.10 ± 0.00	1.86 ± 0.22^*∗*^	0.055 ± 0.0065	—
Prealbumin (PA) (mg/L)	Undetected	344.63 ± 68.17^*∗*^	—	—
Alanine aminotransferase (ALT) (U/L)	76.75 ± 24.29	28.39 ± 24.59^*∗*^	4.04 ± 2.57	0.042
Aspartate aminotransferase (AST) (U/L)	439.31 ± 110.57	34.61 ± 12.31^*∗*^	13.71 ± 4.55	−0.043
Alkaline phosphatase (AKP) (U/L)	311.33 ± 337.10	79.61 ± 19.65^*∗*^	4.07 ± 4.24	0.107
Gamma-glutamyl transpeptidase (GGT) (U/L)	12133.33 ± 4278.56	36.31 ± 34.96^*∗*^	543.54 ± 344.21	0.006
Lactate dehydrogenase (LDH) (U/L)	2427.08 ± 724.34	172.11 ± 26.17^*∗*^	14.32 ± 4.54	0.115
Adenosine deaminase (ADA) (U/L)	2.92 ± 1.11	9.58 ± 2.31^*∗*^	0.31 ± 0.12	0.109
Urea (Ur) (mmol/L)	24.00 ± 3.81	5.58 ± 1.20^*∗*^	4.49 ± 1.23	0.185
Creatinine (Cr) (*μ*mol/L)	505.61 ± 147.19	82.77 ± 6.97^*∗*^	6.15 ± 1.86	−0.011
Uric acid (UA) (*μ*mol/L)	383.14 ± 105.44	363.56 ± 74.61	1.07 ± 0.25	0.513^△^
Glucose (Glu) (mmol/L)	0.073 ± 0.095	5.30 ± 0.60^*∗*^	0.0147 ± 0.20	−0.356^#^
Triglyceride (TG) (mmol/L)	0.12 ± 0.11	1.56 ± 0.98^*∗*^	0.11 ± 0.13	−0.168
Total cholesterol (TC) (mmol/L)	0.90 ± 0.50	4.92 ± 1.00^*∗*^	0.20 ± 0.13	−0.328
Creatine kinase (CK) (U/L)	1725.56 ± 1730.48	128.67 ± 41.22^*∗*^	15.37 ± 19.79	−0.111
Alpha-hydroxybutyrate dehydrogenase (*α*HBDH) (U/L)	2898.61 ± 1506.08	122.06 ± 20.09^*∗*^	24.06 ± 12.02	0.157
Potassium (K^+^) (mmol/L)	24.06 ± 5.82	4.10 ± 0.29^*∗*^	5.92 ± 1.58	−0.144
Sodium (Na^+^) (mmol/L)	93.78 ± 9.66	142.73 ± 1.52^*∗*^	0.66 ± 0.069	−0.112
Chlorine (Cl^−^) (mmol/L)	56.54 ± 11.37	101.86 ± 1.44^*∗*^	0.56 ± 0.11	−0.087
Calcium (Ca) (mmol/L)	8.47 ± 2.86	2.37 ± 0.10^*∗*^	3.59 ± 1.24	−0.050
Magnesium (Mg) (mmol/L)	6.70 ± 2.62	0.76 ± 0.052^*∗*^	8.92 ± 3.51	−0.105
Phosphorus (P) (mmol/L)	24.89 ± 7.16	1.08 ± 0.20^*∗*^	23.73 ± 8.11	−0.065
High-sensitive C-reactive protein (hsCRP) (mg/L)	0.066 ± 0.064	2.05 ± 4.78^*∗*^	0.097 ± 0.091	0.861^△^

The data were presented as mean ± standard deviation. ^*∗*^ *P* < 0.05 versus the corresponding parameter in seminal plasma. ^#^ *P* < 0.05 and ^△^ *P* < 0.01 for the correlation of the same parameter in seminal plasma and serum.
